# Supplementary material for: Miniaturized enantioselective tubular devices for the electromechanical wireless separation of chiral analytes
Source: Chem. 2024 Feb 8;10(2):660–74. doi: 10.1016/j.chempr.2023.11.001 (PMC10857812; doi:10.1016/j.chempr.2023.11.001)
Supplement: Document S1. Figures S1–S8 [file mmc1.pdf]

Chem, Volume *10*

Supplemental information

Miniaturized enantioselective tubular devices for the electromechanical wireless separation of chiral analytes

Sara Grecchi, Gerardo Salinas, Roberto Cirilli, Tiziana Benincori, Sara Ghirardi, Alexander Kuhn, and Serena Arnaboldi

Supplemental Information

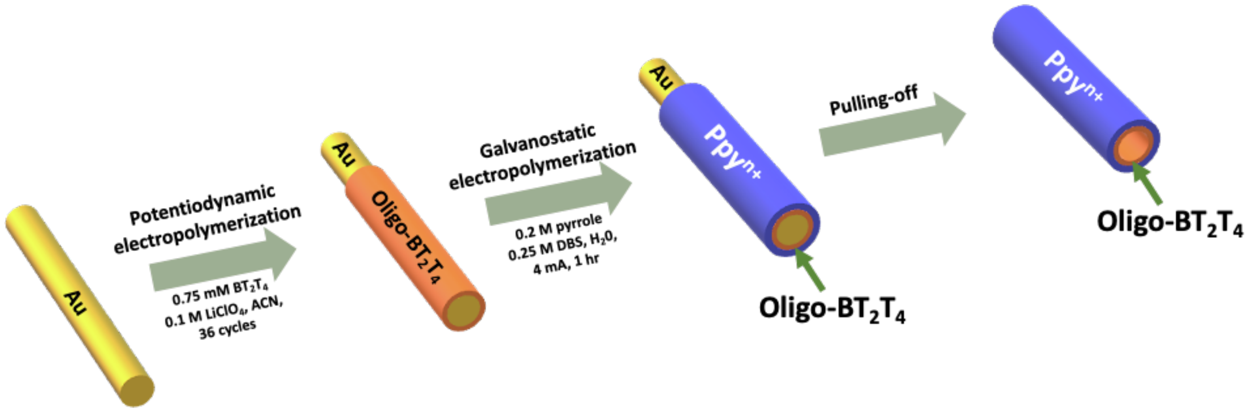

Figure S1. **Schematic illustration of the design of enantioselective tubes.** Schematic illustration of the fabrication steps of the polypyrrole-chiral oligomer tube.

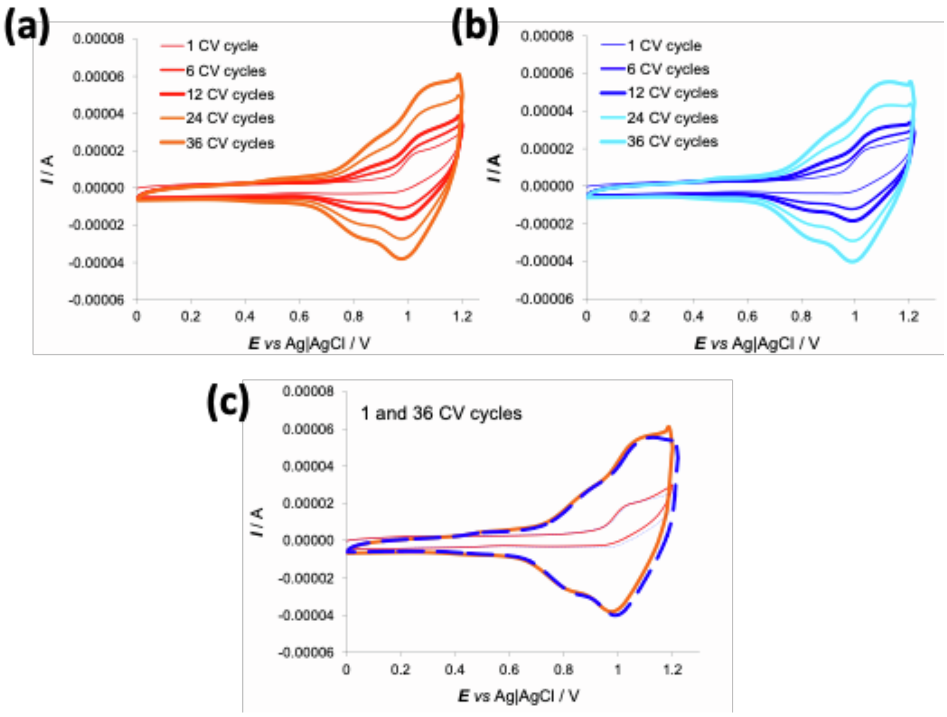

Figure S2. Electrooligomerization of (*S*)- and (*R*)-BT_2T_4. Potentiodynamic electrooligomerization of (a) (*S*)- and (b) (*R*)-BT_2T_4, in red and blue respectively (0. 75 mM in ACN + 0.1 M $LiClO_4$) on a GC electrode by potential cycling with 0.2 V/s. (c) Combined potentiodynamic plots of the 1st and 36th cycle of each monomer.

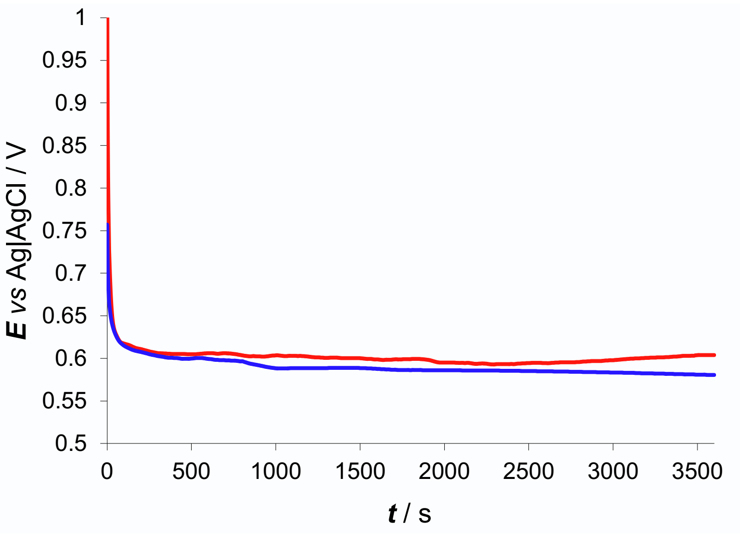

Figure S3. Galvanostatic electropolymerization of pyrrole on a gold wire modified with oligo-(*S*)- or (*R*)-BT$_2$T$_4$. Galvanostatic electropolymerization of pyrrole on a gold wire modified with oligo-(*S*)- or (*R*)-BT$_2$T$_4$ (in red and blue respectively), 400 µA, 3600 s.

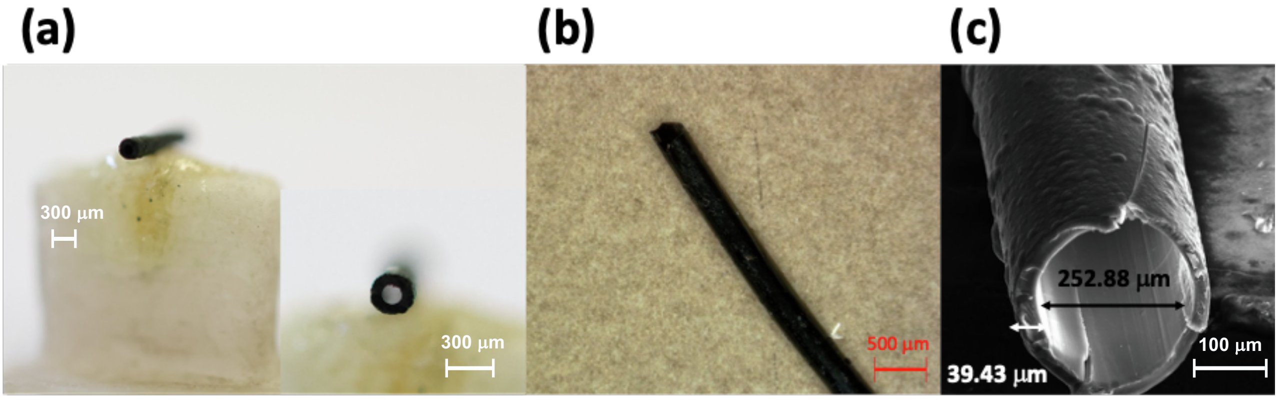

Figure S4. Optical characterization. Optical pictures of the soft tubes; (a) front view when positioned at the top of a plastic holder (scale bars 300 µm) and (b) top view (scale bar 500 µm). (c) SEM micrograph of the front view of one extremity of an unmodified tube. Scale bar 100 µm.

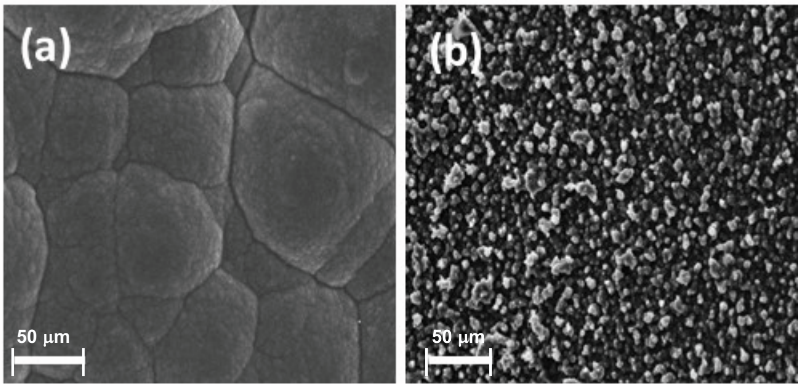

Figure S5. Morphological characterization. SEM micrographs of the a) external part and b) internal part of the tube, obtained cutting the functionalized tube along its main axis. Scale bars 50 µm.

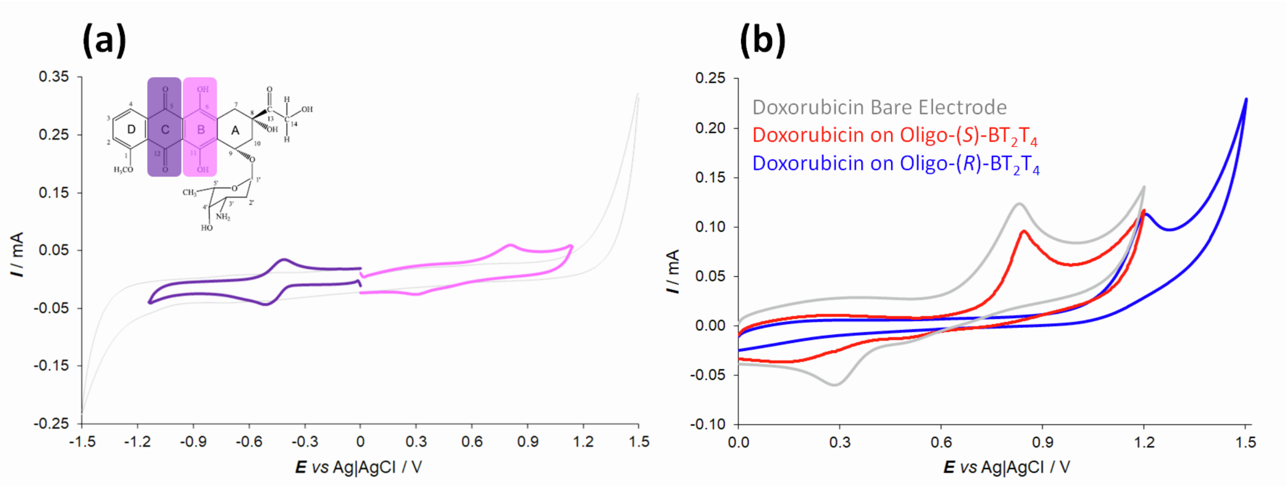

Figure S6. **Electrochemical characterization and enantioselectivity tests of doxorubicin.** (a) First oxidation and reduction peaks (right and left, respectively) of doxorubicin (1 mM in commercial buffer pH 4) on a GC electrode, at 0.2 V s^{-1} potential scan rate. The grey line stands for the background (commercial buffer pH4) measured under the same conditions. Oxidative and reductive half cycles are considered separately to avoid electrode poisoning by electron transfer products. (b) Enantioselectivity tests carried out in a pH 4 buffer solution containing 2 mM doxorubicin with oligo-(*S*)-BT_2T_4 or oligo-(*R*)-BT_2T_4 potentiodynamically deposited on a GC electrode. In grey, the doxorubicin signal recorded with a bare GC electrode.

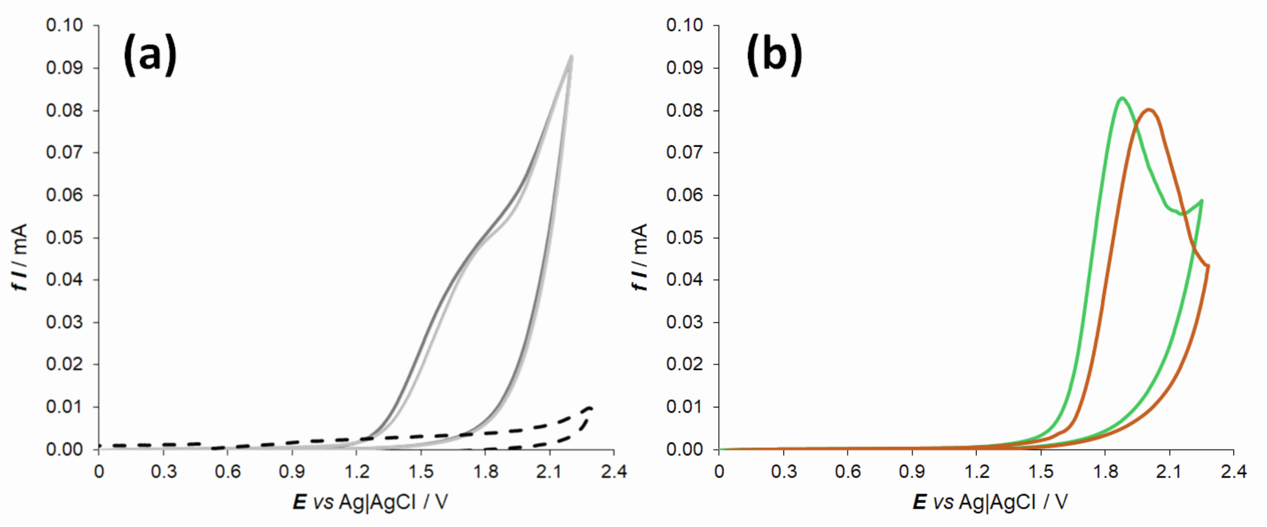

Figure S7. **Enantioselectivity test with Carvone on (*R*)-oligo-BT$_2$T$_4$.** (a) CV patterns recorded on a GC bare electrode at 50 mV/s potential scan rate of (*S*)-(+)- and (*R*)-(-)-carvone. The dotted black line stands for the background recorded in pH 4 buffer solution + EtOH. (b) Enantioselectivity tests in pH 4 buffer solution + EtOH at 50 mV/s potential scan rate of (*S*)-(+)- and (*R*)-(-)-carvone (2 mM, brown and green lines, respectively) on a GC electrode modified with oligo-(*R*)-BT$_2$T$_4$.

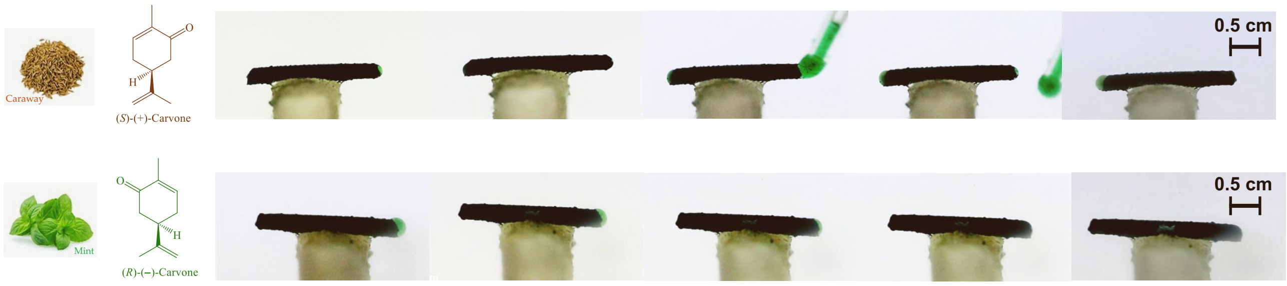

Figure S8. **Enantioselective bipolar electrochemistry experiments with Carvone on (*R*)-oligo-BT$_2$T$_4$.** Specular enantioselective bipolar electrochemistry experiments with (*S*)- and (*R*)-carvone (2 mM) dissolved in BMIMTFSI and chiral tubes modified with oligo-(*R*)-BT$_2$T$_4$. The total time for each acquisition was 10 minutes, optical pictures were taken every 2 minutes. Scale bars 0.5 cm.
