# Supplementary material for: Miniaturized enantioselective tubular devices for the electromechanical wireless separation of chiral analytes
Source: Chem. 2024 Feb 8;10(2):660–74. doi: 10.1016/j.chempr.2023.11.001 (PMC10857812; doi:10.1016/j.chempr.2023.11.001)
Supplement: Document S2. Article plus supplemental information [file mmc14.pdf]

Chem

CellPress
OPEN ACCESS

Article

Miniaturized enantioselective tubular devices for the electromechanical wireless separation of chiral analytes

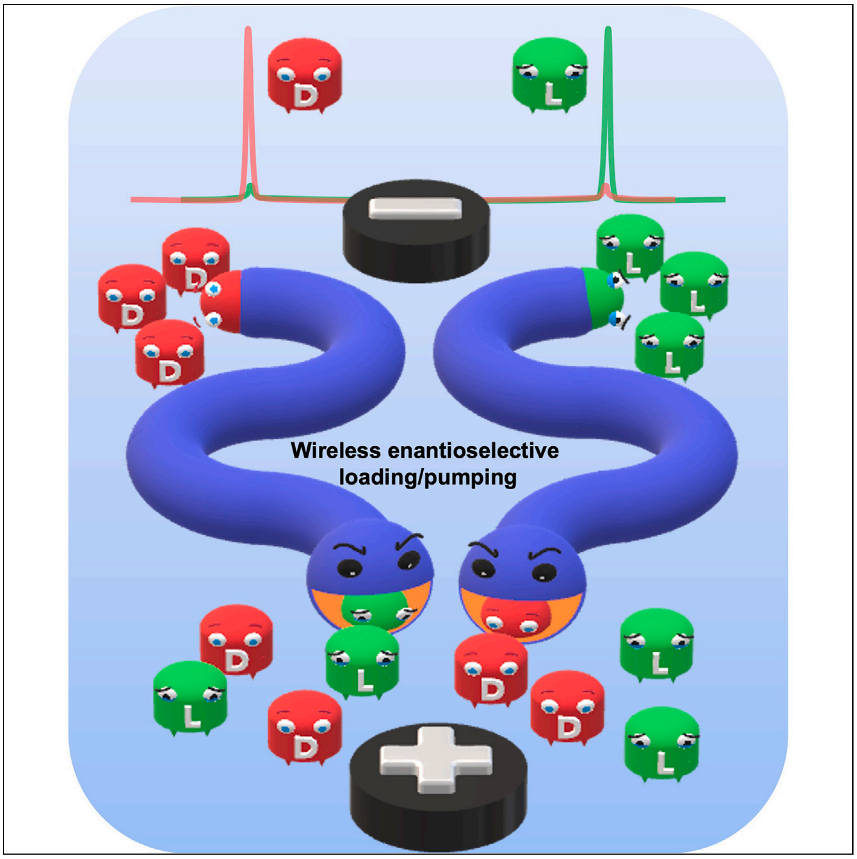

The difference in retention time between the antipodes of a chiral molecule inside the tubes opens the way to new soft systems applicable in the field of miniaturized wireless chiral separation.

Sara Grecchi, Gerardo Salinas, Roberto Cirilli, Tiziana Benincori, Sara Ghirardi, Alexander Kuhn, Serena Arnaboldi

serena.arnaboldi@unimi.it

Highlights

Wireless electropumping for the slow release of chiral analytes

Enantioselective soft objects for the towing of chemically different analytes

Enantioselective wireless microreactors for chiral separation

Grecchi et al., Chem *10*, 660–674
February 8, 2024 © 2023 The Author(s).
Published by Elsevier Inc.
https://doi.org/10.1016/j.chempr.2023.11.001

Chem

Article

Miniaturized enantioselective tubular devices for the electromechanical wireless separation of chiral analytes

Sara Grecchi,[1] Gerardo Salinas,[2] Roberto Cirilli,[3] Tiziana Benincori,[4] Sara Ghirardi,[4] Alexander Kuhn,[2] and Serena Arnaboldi[1,5,*]

SUMMARY

Chirality plays a crucial role in different research fields, ranging from fundamental physico-chemistry to applied aspects in materials science and medicine. In this context, enantioselective loading and pumping of chiral analytes for analysis, separation, and cargo delivery applications is an interesting scientific challenge. Herein, we introduce artificial chiral soft electromechanical pumps based on a bi-layer film built up by electrodepositing polypyrrole and an inherently chiral conducting oligomer at its internal surface. The enantioselective device can be driven by bipolar electrochemistry to act as a pump, allowing the selective loading and separation of different chiral analytes injected as pure enantiomers and in racemic form (i.e., doxorubicin, a chemotherapy drug, limonene, carvone, and a chiral ferrocene). The synergy between wireless electromechanical actuation and inherent enantiodiscrimination features makes these actuators excellent candidates for the controlled handling of chiral molecules in the frame of potential applications ranging from analysis to drug delivery.

INTRODUCTION

Enantiodiscrimination of chiral molecules is an interesting topic in different fields of research, such as chemistry, physics, and medicine.[1,2] Especially in the pharmaceutical industry, the stereochemical configuration of a specific chiral drug can play a crucial role,[3] since, in the human body, enantiomers can exhibit similar, different, or opposite physiological effects. Thus, enantioselective recognition, separation, synthesis, and delivery of chiral drugs are highly desired.[4] For example, ketoprofen, a nonsteroidal anti-inflammatory drug with analgesic and antipyretic effects, shows a therapeutically relevant response only for the (*S*)-enantiomer.[5] However, since the two antipodes of chiral analytes present the same physico-chemical properties, discrimination or separation has been mainly achieved by using high-performance liquid chromatographic or electro-migration methods, which require expensive instrumentation and sophisticated time-consuming procedures.[6,7] In this context, electrochemistry is an interesting alternative to such methods due to its simplicity, low-cost, high sensitivity, and straightforward signal transduction.[8] Recently, a variety of electrochemical approaches for chiral recognition and asymmetric synthesis have been developed, taking advantage of different enantioselective electrode/electrolyte interfaces.[9–12]

Among those, inherently chiral molecular materials are a powerful ingredient for the transduction of chiral information.[9,13] The outstanding enantiorecognition of such

THE BIGGER PICTURE

Chirality plays an important role in multiple fields of science, such as chemistry, biology, or medicine. Although enantioselective separation of chiral molecules, induced by external stimuli, has been well established, the development of miniaturized chiral soft systems able to quickly and wirelessly distinguish such molecules still faces great challenges. The synergy between the mechanical properties and the enantioselectivity of conjugated polymers powered by bipolar electrochemistry (BPE) could lead to so far unexplored effects. The here-prepared soft tubes could mimic chiral columns generally used in high-performance liquid chromatography (HPLC). Furthermore, the wireless mode, an intrinsic feature of BPE, could allow the separation of chiral molecules, avoiding the physical connection to an HPLC apparatus, in a cheap and fast manner. The whole system would be miniaturized, envisioning the possibility of using these devices for wireless chiral separation when low-cost, fast, and *in situ* analyses are required.

 © 2023 The Author(s). Published by Elsevier Inc.
This is an open access article under the CC BY license (http://creativecommons.org/licenses/by/4.0/).

CellPress
OPEN ACCESS

conjugated systems is based on the energetically favored diastereomeric interactions between the inherent chiral surface and a specific enantiomer in solution. This results in a neat separation of the antipodes of a given electroactive chiral molecule in terms of peak potential, which allows to selectively activate only one of the two enantiomers at a given applied potential.[14–17] Recently, the outstanding properties of inherently chiral materials were successfully combined with bipolar electrochemistry (BPE), resulting in interesting applications, e.g., the transduction of chiral information from the molecular level into macroscopic enantioselective motion.[17] BPE is an interesting approach to generate in a wireless manner an asymmetric electroactivity on conducting objects.[18–23] In brief, by applying an external electric field (ϵ), a polarization potential difference (ΔV) is generated at the extremities of a conducting object positioned somewhere in the solution. In the presence of electroactive species, and when the ΔV exceeds a thermodynamic threshold potential (ΔV_{min}), oxidation and reduction are triggered at each extremity of the so-called bipolar electrode (BE).[24,25] In particular, the possible wireless control of the asymmetric reactivity on a BE, in synergy with the electroactivity, tuneability, and processability of conducting polymers, opens up new and so far unexplored possibilities in the field of chemically responsive soft materials.[26,27]

In this context, BPE can be especially used as an interesting alternative for the efficient stimulation of multi-responsive electropumping systems since its efficiency is directly linked to the physico-chemical interactions between the dynamic object and the analyte of interest. Theoretically, the wireless nature of BPE is its main advantage, in comparison with already proposed responsive pumping approaches,[26,28–34] for which a physical connection to an external power supply is required, thus limiting their potential applications, especially in the case of miniaturized systems. Although several micro-fluidic pumping strategies,[29] based on different multi-stimuli responsive materials, have been developed, the precise control of flow rate is still challenging. Thus, simple, efficient, and selective loading and pumping mechanisms are highly desired. Pumping systems driven by electric fields, compared with other external stimuli, are an interesting approach to control the rate and direction of the flow. However, once again, electrically driven soft pumps are still limited by the essential requirement of an electric contact in order to trigger the actuation.[30–32] Herein, we designed an enantioselective soft polymer tube activated in a wireless mode by means of BPE. The wireless enantioselective loading/pumping devices were designed as a free-standing bi-layer tube composed of a polypyrrole (Ppy) chassis internally functionalized with an enantiopure oligo 2,2′-bis[2-(5,2′-bithienyl)]-3,3′-bithianaphthene, named BT_2T_4 (Figure 1A). It has been demonstrated that the chirality of such conjugated molecules is fully transferred from the monomer to the electrochemically produced oligomers.[35–37] The synergy between the pumping mechanism of polypyrrole via BPE and the outstanding enantiorecognition capability of the inherently chiral surfaces leads to a selective loading or pumping of only one enantiomer of a chiral probe via favorable or unfavorable diastereomeric interactions. As a proof of concept, the enantioselective loading/pumping with the designed soft tubes was tested with different chiral probes, i.e., doxorubicin, limonene, and carvone, due to their importance in the pharmaceutical industry. Furthermore, the highly efficient enantioseparation of the here-proposed approach was evaluated by injecting racemates of two chiral analytes with uncorrelated chemical structures. The released fractions, collected during the electromechanically induced loading/pumping, were then analyzed by chiral high-performance liquid chromatography (HPLC) to corroborate their enantiomeric purity and the separation efficiency of these systems.

[1]Dipartimento di Chimica, Università degli Studi di Milano, Via Golgi 19, 20133 Milan, Italy

[2]University of Bordeaux, CNRS, Bordeaux INP, ISM, UMR 5255, 33607 Pessac, France

[3]Centro Nazionale per il Controllo e la Valutazione dei Farmaci, Istituto Superiore di Sanità, Viale Regina Elena, 299, 00161 Rome, Italy

[4]Dipartimento di Scienza e Alta Tecnologia, Università degli Studi dell'Insubria, Via Valleggio 11, 22100 Como, Italy

[5]Lead contact

*Correspondence: serena.arnaboldi@unimi.it

https://doi.org/10.1016/j.chempr.2023.11.001

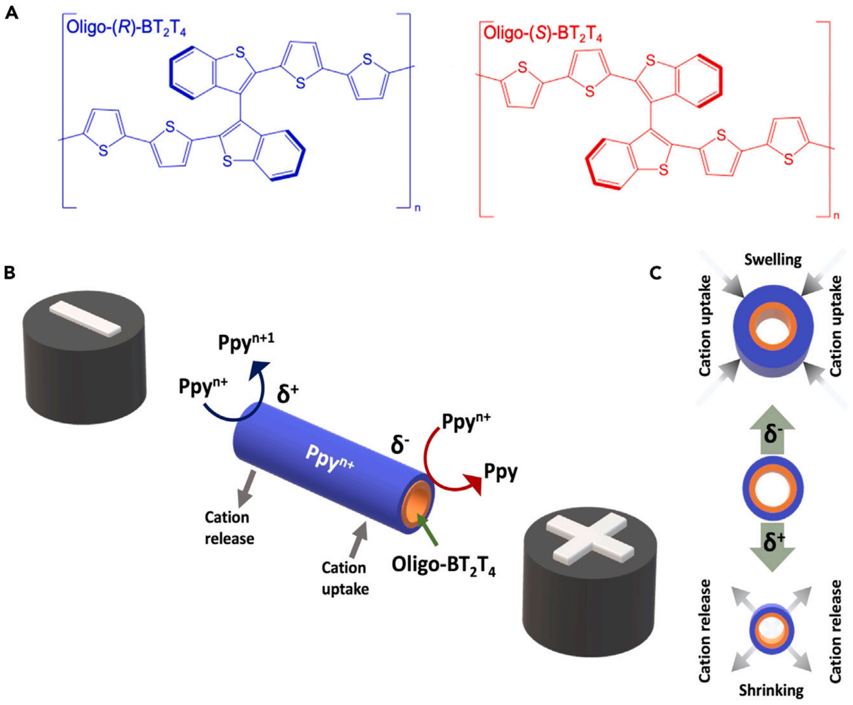

Figure 1. Chemical structures of the enantiopure inherently chiral monomers and schematic illustration of the bipolar setup

(A) Chemical structures of enantiopure oligo-(*R*)- and oligo-(*S*)-BT$_2$T$_4$ (blue and red, respectively).

(B) Schematic illustration of the bipolar setup used for the wireless enantioselective loading/pumping with a representation of the asymmetric polarization, the associated electrochemical reactions, and the induced cation exchange.

(C) Schematic illustration of the asymmetric swelling and shrinking process induced by the electric field, with a representation of the cation exchange. The orange and blue parts symbolize the oligo-BT$_2$T$_4$ and the Ppy films, respectively.

RESULTS

Design of the wireless enantioselective loading/pumping device

The soft tubular devices were designed by following a two-step approach. First, potentiodynamic electropolymerization of the corresponding oligo-BT$_2$T$_4$ on the surface of a gold wire was carried out, followed by the galvanostatic deposition of polypyrrole on the hybrid Au/oligo-BT$_2$T$_4$ surface (Figure S1). The electrochemical growth of both enantiomers of BT$_2$T$_4$ shows the characteristic current increase at potentials beyond the oxidation of the monomer, which indicates the efficient modification of the Au surface (Figures S2A and S2B). Furthermore, the final anodic and cathodic currents are similar for both enantiomers, indicating that the obtained films exhibit theoretically a similar thickness (Figure S2C). The potential transient recorded during the electropolymerization of pyrrole presents a relatively constant value, around ≈ + 0.6 V vs. Ag/AgCl, during the whole deposition experiment, characteristic of the oxidation potential of pyrrole (Figure S3). Subsequently, the modified electrode was dipped in acetone for 15 min, and afterward, the gold template was removed mechanically. Optical pictures of the as-synthesized polymer tube indicate an inner diameter of around ≈ 253 μm, which is consistent with the diameter of the template electrode (Figures S4A–S4C). In addition, a thickness of ≈ 40 μm was measured for the Ppy walls, which leads to an outer diameter of 293 μm. Scanning electron microscopy (SEM) images show a rough morphology for the outer layer of the tube, whereas a slightly smoother surface is present in the inner part of the

unmodified device (Figure S4C). To prove the oligomer functionalization of the internal part of the Ppy tube, the hybrid chiral object was cut along its main axis, and SEM analysis of the two surfaces was carried out. As can be seen in Figure S5, two completely different morphologies were obtained—the characteristic rough external face of Ppy and a granular surface for the inner chiral part (Figures S5A and S5B, respectively). These SEM analyses are in agreement with the ones obtained in previous studies, where free-standing polypyrrole strips were functionalized with oligo-BT_2T_4.[15,16]

Electrochemical characterization and enantioselectivity tests of doxorubicin

The first chiral probe was [5,12-naphthacenedione, 10-[(3-amino-2, 3, 6-trideoxy-alpha-L-lyxo-hexopyranosyl)oxy]-7, 8, 9, 10-tetrahydro-6, 8, 11-trihydroxy-8-(hydroxylacetyl)-1-methoxy-, hydrochloride(8S-cis)], commercially known as doxorubicin hydrochloride, which is the most important member of the family of anthracycline antibiotics, particularly used for chemotherapeutic applications and its antineoplastic action.[38] However, it is responsible for many undesired side effects, ranging from delayed and insidious cardiomyopathy to irreversible heart failure. The potentiodynamic characterization of doxorubicin in a buffer solution (pH 4) on a glassy carbon (GC) electrode exhibits two peaks, around −0.5 V vs. Ag/AgCl and 0.8 V vs. Ag/AgCl, associated with the reduction of the 5,12-diquinone group and the oxidation of the 6,11-dihidroquinone functionality, respectively (Figure S6A).[39–42]

In order to verify the enantiorecognition capability of the inherently chiral films toward doxorubicin, a GC electrode modified with oligo-(*R*)- or (*S*)-BT_2T_4 films was used. The enantiopure oligomeric films were obtained by following the same procedure as the one described above for the electropolymerization of BT_2T_4. Afterward, the two antipodes of the chiral film were tested independently in a buffer solution (at pH 4) containing 2 mM of doxorubicin by means of cyclic voltammetry. Under these conditions, the signal related to the oxidation of the chiral molecule at the surface of the oligo-(*S*) electrode appears at a similar potential value compared with the one obtained with the bare GC electrode (0.8 V vs. Ag/AgCl), whereas with the oligo-(*R*) surface, a shift of 400 mV toward higher anodic values was obtained (Figure S6B). This peak separation between the (*S*)- and (*R*)-modified electrodes indicates an inhibition of the electron transfer caused by an unfavorable diastereomeric interaction.

Wireless enantioselective loading and pumping

After demonstrating the enantioselectivity of the inherently chiral oligomers by means of conventional electrochemistry, we have studied the wireless enantioselective loading and pumping mechanism. At first, a 1-cm-long soft tube was immobilized on an inert support and placed at the bottom of a bipolar cell containing a 0.2 M $LiClO_4$, pH 4 buffer solution, maintaining a constant distance of 5 cm between the feeder electrodes. As stated above, the wireless enantioselective loading/pumping effect takes advantage of the synergy between the electromechanical properties of Ppy and the outstanding enantiorecognition capability of the inherently chiral oligomer. By applying an electric field (ϵ) between the two feeder electrodes, an asymmetric polarization is induced along the free-standing tube (Figure 1B).[43] When a high enough ϵ is applied, oxidation and reduction of the doped Ppy take place at the δ^+ and δ^- poles, respectively (Figure 1B). Commonly, such redox reactions are accompanied by the release and uptake of cations at the anodic and cathodic extremities of the device, respectively, in order to maintain electroneutrality (Figure 1C). This charge-compensating mechanism leads to local swelling and

shrinkage of the extremities of the tube. However, the electromechanical pumping is associated with changes in the inner diameter of the tube; thus, the swelling and shrinking processes cause a decrease and increase in the inner diameter, respectively.[43] Such an asymmetric change of the inner diameter induces a difference in pressure at each extremity of the tube which, according to the Bernoulli principle, triggers a unidirectional flow of liquid. It is important to highlight that the speed and reversibility of the electrochemically induced flux are strongly related to the applied electric field, since higher ε values lead to a faster ion exchange, causing a more pronounced asymmetric change of the inner diameter. Thus, low ε values are required in order to generate a continuous and reversible unidirectional flow of liquid induced by a slow ion exchange. Considering that the ΔV_{min} value that triggers the oxidation and reduction of doped Ppy is found to be around 1.6 V,[44] an ε below 1.6 V/cm is needed to induce a slow ion exchange in a 1 cm Ppy tube. In order to ensure the electromechanical unidirectional flow of liquid, a ε of 1.4 V/cm was applied. Such a value is considerably smaller than the one used to induce electromechanical pumping by alternating current presented in a previous work.[43] In addition, the selected electric field allows for minimizing the risk of oxidization of the chiral analyte during the electromechanical pumping since the oxidation potential values of Ppy and doxorubicin are both really close in the electrochemical window. Nonetheless, it is possible to assume that a small fraction of the electrons that flow from the anode to the cathode of the soft tube originate from the oxidation of doxorubicin. However, the electromechanical tube contraction is not related to any kind of chiral interaction involving just the Ppy chassis. The presence of the oligo-BT_2T_4, deposited in the internal part of the tube, impacts the loading and pumping of the chiral analyte, resulting in a stereoselective retention or release of the probe enantiomers. Thus, a favorable diastereomeric interaction should trigger a loading effect (Figure 2A, upper image) that increases the retention time of the probe inside the tube, whereas an unfavorable diastereomeric interaction would allow the chiral probe to pass through the tube and be more easily released during the electromechanical pumping (Figure 2A, bottom image). In order to corroborate this hypothesis, two tubes, modified at the inner part with either oligo-(*R*)- or oligo-(*S*)-BT_2T_4, were tested under a constant electric field value of 1.4 V/cm. The enantioselective BPE experiments were carried out by dissolving doxorubicin in an achiral commercial ionic liquid, 1-butyl-3-methylimidazolium bis(trifluoromethylsulfonyl)imide (BMIMTFSI). The ionic liquid was used to avoid the spontaneous diffusion of the probe in the aqueous medium. No colorant was added to visualize the enantioselective loading/pumping effect since doxorubicin has a characteristic red color. At first, in the absence of an electric field, the analyte mixture is not captured by the soft tube (Video S1), which allows us to rule out a capillarity effect as the driving force of the pumping. Under a constant electric field (1.4 V/cm), by using the oligo-(*S*)-modified soft tube, the analyte mixture is captured due to the electromechanical unidirectional flow of liquid and remains trapped, caused by a favorable diastereomeric interaction (Figure 2B, top row; Video S2 left side). On the contrary, while using the oligo-(*R*)-modified device, the doxorubicin mixture flows from the cathodic to the anodic extremity of the tube (Figure 2B bottom; Video S2 right side). The observed enantioselectivity is in good agreement with the potentiodynamic measurements, thus corroborating the effect of the diastereomeric interactions occurring at the inner part of the tube. Furthermore, by applying a constant electric field for a long period of time (around 20–25 min), the doxorubicin/BMIMTFSI mixture is expulsed from the oligo-(*S*)-modified soft tube (Video S3). It is important to highlight that the electrochemically driven loading and pumping mechanism benefits not only from the mechanical properties of Ppy, but also from the interactions between the inherent chiral oligomer present at the inner part of the tube and the chiral analyte.

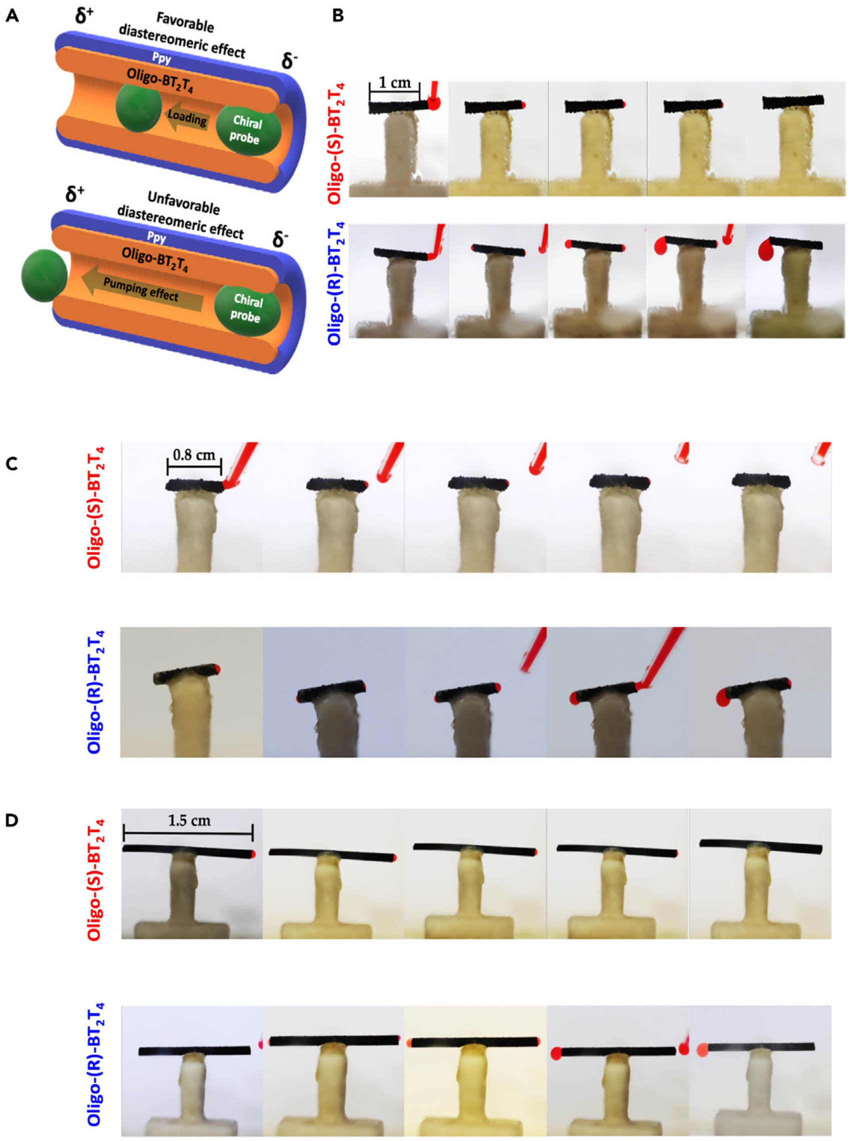

Figure 2. Wireless enantioselective loading and release of doxorubicin using tubes with different lengths

(A) Schematic illustration of the loading/pumping mechanism driven by the favorable (top) and unfavorable (bottom) diastereomeric interactions between the chiral probe and the oligo-BT_2T_4. The orange and blue parts symbolize the oligo-BT_2T_4 and the Ppy film, respectively, whereas green represents the chiral probe.

(B–D) (B) Optical pictures of the wireless enantioselective loading/pumping observed with Ppy tubes modified at the internal part with oligo-(*S*)- (top) or oligo-(*R*)-BT_2T_4 (bottom), at a constant electric field value (1.4 V/cm). The global time of the experiment was 10 min and optical pictures were taken every 2 min. Scale bars, 1cm. Optical pictures of the wireless enantioselective loading/pumping obtained with Ppy tubes with a length of (C) 0.8 cm (scale bars, 0.8 cm) and (D) 1.5 cm (scale bars, 1.5 cm) modified in the internal part with oligo-(*S*)- (top row) or oligo-(*R*)-BT_2T_4 (bottom row) at a constant electric field value (1.7 V/cm in C and 1.2 V/cm in D). The global time of the experiment was 10 min, with pictures taken every 2 min.

This was demonstrated by studying the electromechanical pumping of an unmodified Ppy tube, indicating the absence of an inherent chiral oligomer. As it can be seen, by applying a constant electric field (1.4 V/cm), it is not possible to induce

the electromechanical loading of the analyte (Video S4). Thus, the presence of a chiral environment at the inner part of the device facilitates the loading and subsequent expulsion of the chiral probe due to the favorable and unfavorable diastereomeric interactions.

However, due to the complexity of the physico-chemical system, a deeper analysis of the loading/pumping mechanism is required. In order to provide a deeper insight, we studied the effect of the nature of the liquid used as the solvent of the chiral probe on the electromechanical unidirectional flux. For this set of experiments, we used chlorinated paraffin as an alternative (CAS: 85422-92-0), and we tested the enantioselective loading/pumping mechanism at a constant electric field (1.5 V/cm). Once again, by using the oligo-(*S*)- modified device, the chiral mixture is captured (and released only after a long period of time), whereas using the oligo-(*R*)- modified soft tube, the doxorubicin mixture flows almost immediately from the cathodic to the anodic extremity (Videos S5 and S6). Since the same enantioselective loading/pumping behavior was obtained in the presence of an ionic (BMIMTFSI) and non-ionic liquid (chlorinated paraffin), it is possible to rule out an electroosmotic mechanism during the unidirectional pumping.

In addition, such an enantioselective electromechanical loading/pumping mechanism can be extended to free-standing modified Ppy tubes with different lengths. In this case, shorter BEs (0.8 cm) require higher electric fields (1.7 V/cm) in comparison to the 1 cm BE, whereas for longer soft tubes (1.5 cm), the necessary electric field decreases (1.2 V/cm). This is in good agreement with the principles of BPE, where the ΔV produced between the extremities of a conducting object, when exposed to the electric field and for a given set of redox reactions, is a function of the length (l) of the device (equation 1).

$$\varepsilon = \Delta V / l \quad \text{(Equation 1)}$$

Although short tubes (0.8 cm) remain mechanically stable after first use, long devices (1.5 cm) exhibit cracks and poor stability after the bipolar experiments. This is associated with additional redox reactions, i.e., overoxidation of the conjugated backbone or more pronounced swelling and shrinking effects. Nonetheless, for all the different polymer tubes, the same enantioselective behavior was observed, i.e., in the case of the matching system, oligo-(*S*)-/doxorubicin, the chiral probe is loaded inside the pump (Figures 2C and 2D top; Videos S7 and S8 left panels for 0.8 cm and 1.5 cm long tubes, respectively), whereas for the mismatching combination, oligo-(*R*)-/doxorubicin, a pumping effect is produced (Figures 2C and 2D bottom; Videos S7 and S8 right panels for 0.8 cm and 1.5 cm long tubes, respectively).

Wireless enantioselective loading and pumping of alternative chiral probes

The general validity of the concept was evaluated by using alternative chiral probes, i.e., limonene and carvone. These chiral molecules are two monoterpenes that exist in two different enantiomeric configurations. Both enantiomers have well-pronounced flavors and aromas, which allow their possible discrimination through our nasal receptors. In fact, the enantiomers of limonene present a very characteristic smell, lemon and orange for the (*S*)-and (*R*)-enantiomers, respectively. Instead, in the case of carvone, the (*R*)-enantiomer has a mint-type aroma, whereas the (*S*)-isomer, as the key component of caraway, presents an anise-like smell. Furthermore, carvone has multiple biological properties, including antidiabetic, anti-inflammatory, anticancer, neurological, antimicrobial, antiparasitic, antiarthritic, anticonvulsant, and immunomodulatory effects. In pharmaceuticals, limonene is added to help medicinal ointments and creams penetrate the skin.[45,46]

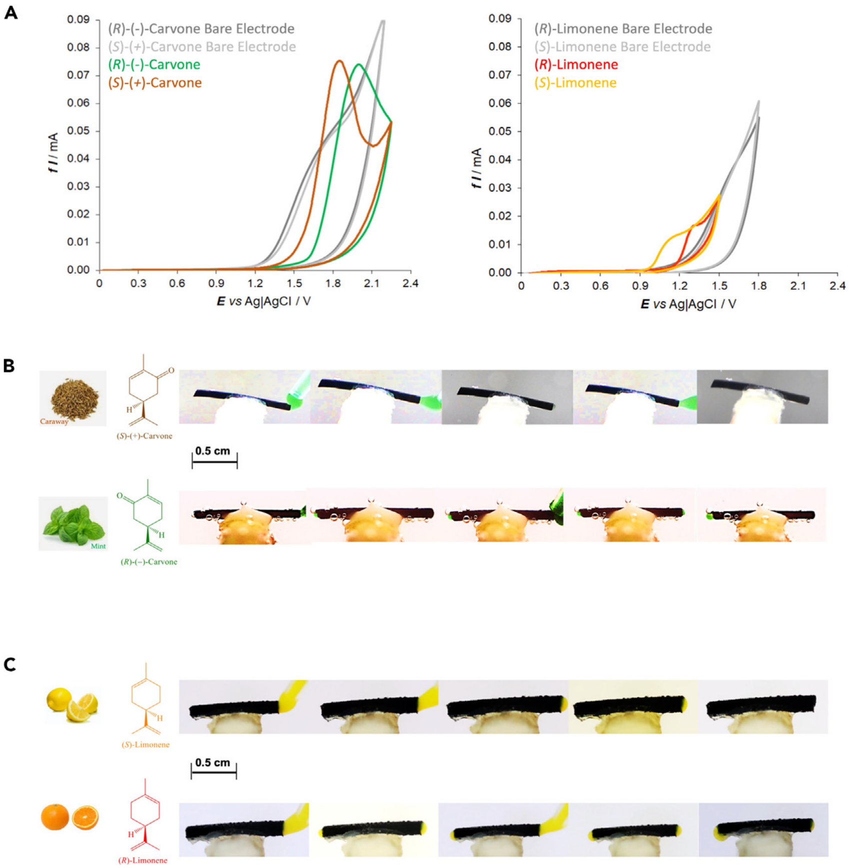

Figure 3. Electrochemical enantiorecognition tests together with the wireless enantioselective loading and pumping of carvone and limonene

(A) Potentiodynamic enantioselectivity tests for (*S*)- and (*R*)-carvone (left plot, orange and green lines, respectively) and (*S*)- and (*R*)-limonene (right plot, yellow and red lines, respectively) obtained on a GC electrode modified with oligo-(*S*)-BT_2T_4 in a 2 mM chiral probe, pH 4 buffer solution + EtOH at 50 mV/s scan rate. The potentiodynamic profiles in gray were recorded on a bare electrode.

(B and C) Optical pictures of the wireless enantioselective loading/pumping experiments, carried out with Ppy tubes modified in the internal part with the oligo-(*S*)-BT_2T_4, at a constant electric field value (1.4 V/cm), recorded for (b) (*S*)- and (*R*)-carvone (2 mM) and (C) (*S*)- and (*R*)-limonene (2 mM) dissolved in BMIMTFSI. The duration of the experiments was 10 min, with pictures taken every 2 min. Scale bars, 0.5 cm.

Due to their interesting properties, these molecules were used to verify the versatile nature of the approach proposed here. As for the above-mentioned experiments, at first, the enantiomers of both compounds were tested on a bare GC electrode (in pH 4 buffer solution + 100 μL of EtOH to increase the solubility of the chiral compounds). The potentiodynamic profiles of the enantiomers of carvone and limonene exhibit a broad shoulder around 1.64 V vs. Ag/AgCl on a bare GC electrode (Figure 3A, left and right). Since such a signal is absent on the potentiodynamic profile of the background, it is possible to assume that this current shoulder is associated with the oxidation of the antipodes when they are adsorbed at the carbon surface (Figure S7A). However, under the same experimental conditions and using the oligo-(*S*)- or oligo-(*R*)-BT_2T_4-modified electrodes, better-defined signals were observed for both probe/oligomer combinations with a peak-to-peak difference of around 190 and 180 mV for carvone and limonene, respectively (Figures 3A and S7B). The

interaction between the chiral probe and the oligomer leads to oxidation of the analyte on the film surface, producing well-defined potentiodynamic signals. A similar affinity pattern as with doxorubicin was observed, i.e., the *R*-oligomer film inhibits the oxidation of the *S*-probe of limonene and carvone. Moreover, in the case of carvone, the specular experiment was also performed by depositing on the electrode surface the *S*-oligomer film that inhibits the *R*-probe (Figure S7B).

After this set of experiments, wireless enantioselective loading and pumping were evaluated for both chiral molecules. Once again, the antipodes of each probe were dissolved in BMIMTFSI before the electromechanical measurements. However, since these molecules are colorless, two uncharged dyes were added to the mixtures, PY 1 yellow (2-[(4-methyl-2-nitrophenyl)diazenyl]-3-oxo-N-phenylbutanamide) for limonene and Pg8 green (nitroso iron complex) for carvone, respectively. In this set of experiments, the loading/pumping effect of a 1 cm modified BPE with a constant electric field (1.4 V/cm for both limonene and carvone) in the presence of each chiral probe was evaluated. Such a low electric field value allows the electromechanical unidirectional flow of liquid, avoiding the oxidation of the chiral analyte inside the soft tube. In addition, under these conditions, the used colorants do not exhibit any electroosmotic impact on the pumping mechanism since electric fields above 3.2 V/cm are required to release the ionic liquid loaded with the corresponding colorant (Video S9) from the tube. Using the oligo-(*S*) modified tube, (*S*)-(+)-carvone is loaded, caused by the favorable diastereomeric interactions (Figure 3B, top; Video S10A), whereas the (*R*)-(−)-carvone is pumped from the cathodic to the anodic extremity of the tubular object (Figure 3B, bottom; Video S10B). The specular loading/pumping effect was obtained when using the oligo-(*R*)-modified BE, for which the (*S*)-(+)-carvone was pumped and the (*R*)-(−)-carvone was loaded (Figure S8; Videos S10C and S10D). Finally, the same behavior as in the case of doxorubicin was observed for limonene. When using the oligo-(*S*)-modified tube, only (*S*)-limonene gets loaded (Figure 3C top; Video S11, front and side views), whereas the (*R*)-limonene is pumped along the tube (Figure 3C bottom; Video S12).

Wireless enantioselective separation of racemic solutions

After studying the enantioselective properties of the modified polypyrrole tubes, we decided to explore the separation capability of these devices. Since the diastereomeric interactions (favorable or unfavorable) between the inherently chiral oligomers and the chiral probes allow to selectively retain or release a given antipode, it is possible to assume that by injecting directly a racemate of the chiral analytes, the electromechanical pump can mimic the enantioseparation capabilities of a commercial chromatographic column. In order to corroborate this hypothesis, the enantioseparation of the racemate of carvone was carried out by using an oligo-(*R*)-modified Ppy tube. In this case, the racemic probe was used as such (without further addition of any dye, ionic liquid, or paraffin). Chiral HPLC was used to evaluate the composition of the fractions released from the electromechanical chiral pump. Under a constant electric field (1.4 V/cm), four fractions were collected at different times (4 s, 15 s, 20 min, and 25 min). As can be seen by the chromatograms, the first two released fractions are enriched with the unfavored enantiomer (the (*S*)-carvone) (Figure 4A). On the contrary, for the last two fractions, the highest chromatographic peak corresponds to the (*R*)-antipode, which is retained inside the tubular device for almost 20/25 min (Figure 4A). The enantiomeric excess (ee) values for the first and second fractions are 98% for (*S*)-carvone and 2% for (*R*)-carvone, whereas for the third and fourth fractions, the ee is 3% for the (*S*)-enantiomer and 96% for the (*R*)-enantiomer. This is in good agreement with the potentiodynamic and electromechanical

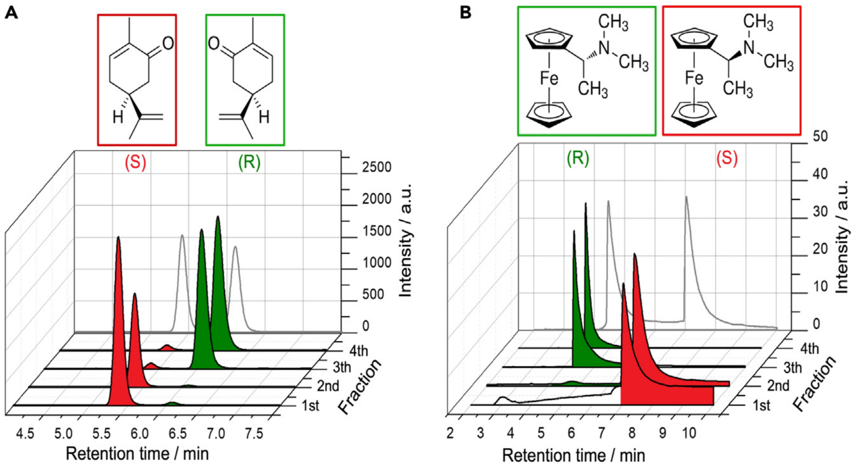

Figure 4. Enantioselective separations of racemic mixtures

(A) Chromatograms of four individual fractions obtained during the electropumping of carvone racemate with a (*R*)-Ppy tube (global time of the experiment 25 min).

(B) Chromatograms of four individual fractions obtained during the electropumping of N,N-dimethyl-1-ferrocenyl-ethylamine racemate with a (*R*)-Ppy tube (global time of the experiment 25 min). The gray chromatograms represent the direct HPLC analysis of the racemic probes before injection into the modified Ppy tube.

enantioselective recognition measurements, since, as stated above, the oligo-(*R*)-device exhibits a favorable diastereomeric interaction with (*R*)-carvone.

Finally, to demonstrate the robustness of the enantioselective separation related to the designed soft pumps, the analysis of the racemate of *N*,*N*-dimethyl-1-ferrocenyl-ethylamine was carried out. In fact, this modified chiral ferrocene presents a completely uncorrelated chemical structure with carvone, limonene, or doxorubicin. Once again, under a constant electric field (1.4 V/cm), four fractions were collected and analyzed by chiral HPLC. The chromatograms of the first two released fractions exhibit mainly a peak corresponding to the (*S*)-antipode, whereas the last two fractions present only the chromatographic peak corresponding to the (*R*)-enantiomer (Figure 4B). Previous potentiodynamic experiments[7] have demonstrated that the (*R*)-oligomer preferentially oxidizes the (*R*)-ferrocenyl probe (and vice versa). Thus, from the sequence shown in Figure 4B, it is evident that the racemate, injected in the modified (*R*)-Ppy tube, is well separated into the two enantiomers. These results clearly demonstrate that the here-presented chiral soft pumps work efficiently, even in the presence of analytes with uncorrelated chemical structures (with high or low steric hindrance) or different chemical characters (aromatic or non-aromatic).

DISCUSSION

In previous work, BPE has been presented as a powerful tool to induce a pumping effect via the electromechanical properties of conducting polymers.[43] Such a system has the main advantage of allowing the wireless induction of the pumping effect without the need for further physical connections that commonly induce rigidity in the system. With the approach described herein, we have introduced additional functionality to wireless electropumping, i.e., enantioselectivity via the internal functionalization of the polypyrrole tubes with inherently chiral oligomers. The outstanding enantiorecognition mechanism that characterizes these oligomers allows them to selectively recognize just one of the antipodes of a chiral analyte at a given applied electric field. The synergy between these two main ingredients, the

electromechanical properties of polypyrrole and the enantiorecognition features of inherent chiral oligomers, leads to two main and so far unexplored effects, namely the enantioselective loading and pumping of chiral analytes. Thus, the inherently chiral soft tubes can load the preferred enantiomer for several minutes and release the other stereoisomer after only a few seconds, depending on the diastereomeric interactions between the oligomers and the analytes. Furthermore, these systems can separate racemic probes into the corresponding antipodes, the latter being obtained with high enantiomeric purity. This concept is generic for electroactive chiral probes since it works equally well for several analytes with complex chemical compositions. In addition, the efficient control of simple experimental parameters, such as the length of the BPE and the distance between the feeder electrodes, allows for optimizing the loading/pumping efficiency. The devices can be recovered and reused multiple times by washing their internal surface, since after the measurements, almost no analyte remains inside the hollow tube. Furthermore, due to the low polarization potential applied along the object, the overoxidation of the film can be ruled out; thus, the initial charged state of the Ppy device can be recovered by simply inverting the polarity of the applied electric field. Although the here-presented findings are the result of a first set of proof-of-concept experiments, they allow imagining that by fine-tuning the physical properties of the polymer tubes, i.e., inner and outer diameters, thickness, shape, and length, it should be possible to modulate their behavior to adapt them to more complex applications, such as macro-, micro-, and nano-devices for slow release functionalities implemented in drug delivery systems. Furthermore, the viscosity of the medium in which the analytes are dissolved guarantees the formation of a well-defined drop that can be considered a microenvironment in which reactions can take place. In particular, by fine-tuning the applied electric field, it is possible that certain types of molecules (easily oxidizable such as doxorubicin), when retained inside the tube, can also be transformed to a certain extent. For this reason, in future experiments, we are planning to perform the enantioselective conversion of a prochiral precursor into its chiral form in a drop of a viscous medium, which is swallowed by the chiral tube. In this case, the drop would act like a microreactor, containing the chiral species generated *in situ* inside the object. As a matter of fact, enantioselective approaches are highly desirable in cargo-towing systems to load and unload selectively active chiral molecules. The difference in retention time between the antipodes of a chiral molecule inside the tube also allows for envisioning the applicability of these devices as miniaturized enantioselective columns for chiral separation. In fact, our device presents multiple advantages in comparison to conventional chiral HPLC, such as the low cost of the equipment and fast separation times, in synergy with its wireless nature and the relatively straightforward tunability of the redox reactions occurring at each extremity of the device.

EXPERIMENTAL PROCEDURES

Resource availability

Lead contact

Further information and requests for resources should be directed to and will be fulfilled by the lead contact, Serena Arnaboldi (serena.arnaboldi@unimi.it).

Materials availability

All materials generated in this study are available from the lead contact without restriction.

Data and code availability

This study did not generate any datasets.

Potentiodynamic measurements of doxorubicin, carvone, and limonene on glassy carbon (GC) electrodes

The three chiral probes were electrochemically characterized by cyclic voltammetry by using a classic three-electrode electrochemical cell (3 cm^3 of solution) coupled with GC (Metrohm, A = 0.033 cm^2), platinum (Pt) wire, and silver/silver chloride (Ag/AgCl) as working (WE), counter (CE), and reference (RE) electrodes, respectively. The three chiral probes in the 1–2 mM concentration range were dissolved in commercial pH 4 buffer solution (Fluka, prepared with citric acid, NaOH, and NaCl), with the addition of 100 μL of ethanol (EtOH) in the case of both carvone and limonene in order to increase their solubility. The enantioselectivity tests were carried out by using the same experimental conditions, but using a GC electrode modified with oligo-(*S*)- or oligo-(*R*)-BT_2T_4 films. Enantiopure oligo-(*S*)- or oligo-(*R*)-BT_2T_4 films were obtained performing 36 potential cycles at a 200 mV/s scan rate, starting from the corresponding enantiopure monomers (0.75 mM fixed concentration) in acetonitrile (ACN) + lithium perchlorate ($LiClO_{4)}$ 0.1 M as the supporting electrolyte. The cell was constituted by GC as the WE, a Pt wire and an Ag/AgCl as CE and RE, respectively.

Synthesis of the enantioselective soft tubes

The soft tubular devices were designed by following a two-step approach. First, the potentiodynamic electropolymerization of the corresponding oligo-BT_2T_4 (0.75 mM in ACN, 36 cycles, v = 200 mV/s) on the surface of a gold wire (Au, d = 0.3 mm) was carried out in a classic three-electrode electrochemical cell, using a Pt wire and an Ag/AgCl as CE and RE, respectively. After the electrooligomerization, the gold wire covered by the enantiopure inherently chiral film was washed with ACN to remove monomer residuals, dried, and used as support for the polypyrrole (Ppy) electrodeposition. The galvanostatic electropolymerization of pyrrole (400 μA for 3,600 s) was performed in a 0.2 M monomer 0.25 M sodium dodecilbenzensulfonate (DBS) aqueous solution. Afterward, the tube, constituted of polypyrrole + oligo-(*S*)- or oligo-(*R*)-BT_2T_4, was mechanically removed from the gold wire (used as the template) after dipping it in acetone for 15 min.

Microscope and SEM images

Microscope images were recorded with a digital Leica DVM6 model. SEM observations were performed with a Tescan Vega model.

Wireless achiral loading/pumping experiments

For the wireless achiral loading/pumping experiments, enantiopure soft tubes based on polypyrrole modified at the inner part with oligo-(*S*)-BT_2T_4 were fixed in the middle of a classic bipolar cell on an inert support. Two graphite feeder electrodes were positioned at the extremities of the cell (5 cm apart). The electrolyte solution was a pH 4 buffer + 0.2 M $LiClO_4$ as supporting electrolyte to provide a sufficient amount of ions for the electromechanical charge compensation mechanism of polypyrrole. In order to visualize the loading/pumping effect, a drop of achiral commercial ionic liquid BMIMTFSI (CAS: 174899-83-3; Aldrich 98%) and drops of (1) pristine BMIMTFSI, or with the addition of (2) PY1 and with (3) Pg8, nitroso iron complex, were approached to one of the holes of the inherently chiral soft pump. All the measurements were carried out in a constant electric field. Experiments were monitored by using a charge-coupled device (CCD) camera (CANON EOS R7, Objective Canon Macro Lens 100 mm 1:2.8). Images were processed with ImageJ software.

Wireless enantioselective loading/pumping experiments

For the wireless enantioselective loading/pumping experiments, enantiopure soft tubes based on polypyrrole modified at the inner part with oligo-(*S*)- or

oligo-(*R*)-BT_2T_4 were fixed at the middle of a classic bipolar cell on an inert support. Two graphite feeder electrodes were positioned at the extremities of the cell (5 cm apart). The electrolyte solution was a pH 4 buffer + 0.2 M $LiClO_4$ as supporting electrolyte to provide a sufficient amount of ions for the electromechanical charge compensation mechanism of polypyrrole. The chiral probes (2 mM) were dissolved (1) in an achiral commercial ionic liquid BMIMTFSI (CAS: 174899-83-3; Aldrich 98%) and (2) in a commercial chlorinated paraffin (CAS: 85422-92-0), which are not soluble in water. For limonene and carvone (colorless liquid), a dye was added to the mixture (PY1 and Pg8, sodium;iron(2+);1-nitrosonaphthalen-2-olate, respectively). However, no dye was added in the case of doxorubicin which shows a natural red color. In order to visualize the loading/pumping effect, a drop of a few microliters of the corresponding chiral mixture was approached to one of the holes of the inherently chiral soft pump. All the measurements were carried out with a constant electric field. Experiments were monitored by using a CCD camera (CANON EOS R7, Objective Canon Macro Lens 100 mm 1:2.8). Images were processed with ImageJ software.

Wireless enantioseparation experiments

For the wireless enantioseparation experiments, enantiopure soft tubes based on polypyrrole, modified at the inner part with oligo-(*R*)-BT_2T_4 were fixed in the middle of a classic bipolar cell on an inert support. Two graphite feeder electrodes were positioned at the extremities of the cell (5 cm apart), containing a pH 4 buffer + 0.2 M $LiClO_4$ solution acting as supporting electrolyte. All the measurements were carried out at a constant electric field of 1.4 V/cm. The racemates of carvone (CAS: 1335436-22-0) and N,N-dimethyl-1-ferrocenyl-ethylamine (50:50 prepared from (*S*)- enantiomer CAS: 31886-57-4 and (*R*)-enantiomer CAS: 19342-01-9) were used as such without further addition of solvents and/or dyes. In two separate experiments, a drop of a few microliters of each racemic probe was approached to the negatively charged side of the inherently chiral soft pump. All the released fractions were collected from the anodic end using a microsyringe and the products were extracted using heptane (3 mL).

Chiral HPLC analysis was carried out with a HPLC equipment (Agilent 1260 Infinity II) coupled with a Daicel CHIRALPAK IG-3 column in isocratic reverse phase conditions. The HPLC analysis of carvone racemate was performed by injecting 1 μL of each heptane solution in the chiral column with ACN/H_2O 50:50 as eluent and a 1 mL/min flow. The photodiode array (PDA) detector was operating at a wavelength of 236 nm. The HPLC analysis of N,N-dimethyl-1-ferrocenyl-ethylamine racemate was carried out by injecting 50 μL of each heptane solution in the chiral column with Hexane/Ethanol/Dichloromethane 99:1:0.1 as the eluent and with a 1 mL/min flow. The PDA detector was tuned to a wavelength of 420 nm. In all cases, both the racemic mixture and each enantiomer were also tested separately under the same conditions in order to compare the enantioseparation results with the starting material and to correctly assign the chromatographic peaks to the corresponding enantiomer.

The ee of the fractions was calculated by using the following equation:

$$Enantiomeric\ excess(ee) = \frac{[(R)E-(S)E]}{[(R)E+(S)E]} x\,100$$

where $(R)E$ and $(S)E$ are the integrated HPLC peak areas of (*R*)- and (*S*)-enantiomers of the analyte under study.

SUPPLEMENTAL INFORMATION

Supplemental information can be found online at https://doi.org/10.1016/j.chempr.2023.11.001.

ACKNOWLEDGMENTS

This work has been funded by the H2020 European Research Council (ERC) under the HORIZON-ERC-2021 work program (grant agreement no. 101040798, ERC Starting grant CHEIR) and under the Horizon 2020 research and innovation program (grant agreement no. 741251, ERC Advanced grant ELECTRA). S.A. is grateful to the students Filippo Malacarne and Massimo Dell'Edera for their contributions in HPLC measurements.

AUTHOR CONTRIBUTIONS

Conceptualization: S.A.; methodology: S. Grecchi, G.S., A.K., and T.B.; investigation: S. Grecchi, G.S., and R.C.; funding acquisition: S.A. and A.K.; project administration: S.A.; supervision: S.A.; writing – original draft: S. Grecchi, G.S., and S.A.; writing – review & editing: S. Grecchi, G.S., S.A., A.K., R.C., T.B., and S. Ghirardi.

DECLARATION OF INTERESTS

The authors declare no competing interests.

INCLUSION AND DIVERSITY

We support inclusive, diverse, and equitable conduct of research.

Received: July 7, 2023
Revised: September 25, 2023
Accepted: November 3, 2023
Published: November 30, 2023

REFERENCES

1. Maier, N.M., Franco, P., and Lindner, W. (2001). Separation. J. Chromatogr. A *906*, 3–33.
2. Okamoto, Y., and Ikai, T. (2008). Chiral HPLC for efficient resolution of enantiomers. Chem. Soc. Rev. *37*, 2593–2608.
3. Nguyen, L.A., He, H., and Pham-Huy, C. (2006). Chiral drugs: an overview. Int. J. Biomed. Sci. *2*, 85–100.
4. Scriba, G.K.E. (2016). Chiral recognition in separation science – an update. J. Chromatogr. A *1467*, 56–78.
5. Cabré, F., Fernández, M.F., Calvo, L., Ferrer, X., García, M.L., and Mauleón, D. (1998). Analgesic, antiinflammatory, and antipyretic effects of S(+)-ketoprofen in vivo. J. Clin. Pharmacol. *38*, 3S–10S.
6. Gumustas, M., Ozkan, S.A., and Chankvetadze, B. (2018). Analytical and preparative scale separation of enantiomers of chiral drugs by chromatography and related methods. Curr. Med. Chem. *25*, 4152–4188.
7. Arnaboldi, S., Grecchi, S., Magni, M., and Mussini, P.R. (2018). Electroactive chiral oligo- and polymer layers for electrochemical enantiorecognition. Curr. Opin. Electrochem. *7*, 188–199.
8. Dincer, C., Bruch, R., Costa-Rama, E., Fernández-Abedul, M.T., Merkoçi, A., Manz, A., Urban, G.A., and Güder, F. (2019). Disposable sensors in diagnostics, food, and environmental monitoring. Adv. Mater. *31*, e1806739.
9. Salinas, G., Niamlaem, M., Kuhn, A., and Arnaboldi, S. (2022). Recent advances in electrochemical transduction of chiral information. Curr. Opin. Colloid Interface Sci *61*, 101626.
10. Niu, X., Yang, X., Li, H., Liu, J., Liu, Z., and Wang, K. (2020). Application of chiral materials in electrochemical sensors. Mikrochim. Acta *187*, 676.
11. Gong, W., Chen, Z., Dong, J., Liu, Y., and Cui, Y. (2022). Chiral metal-organic frameworks. Chem. Rev. *122*, 9078–9144.
12. Wattanakit, C., and Kuhn, A. (2020). Encoding chiral molecular information in metal structures. Chem. Eur. J. *26*, 2993–3003.
13. Arnaboldi, S., Magni, M., and Mussini, P.R. (2018). Enantioselective selectors for chiral electrochemistry and electroanalysis: stereogenic elements and enantioselection performance. Curr. Opin. Electrochem. *8*, 60–72.
14. Arnaboldi, S., Benincori, T., Cirilli, R., Grecchi, S., Santagostini, L., Sannicolò, F., and Mussini, P.R. (2016). "Inherently chiral" thiophene-based electrodes at work: a screening of enantioselection ability toward a series of pharmaceutically relevant phenolic or catecholic amino acids, amino esters, and amine. Anal. Bioanal. Chem. *408*, 7243–7254.
15. Arnaboldi, S., Gupta, B., Benincori, T., Bonetti, G., Cirilli, R., and Kuhn, A. (2020). Absolute chiral recognition with hybrid wireless electrochemical actuators. Anal. Chem. *92*, 10042–10047.
16. Arnaboldi, S., Gupta, B., Benincori, T., Bonetti, G., Cirilli, R., and Kuhn, A. (2020). Large scale chirality transduction with functional molecular materials. Chem. Mater. *32*, 10663–10669.
17. Arnaboldi, S., Salinas, G., Karajić, A., Garrigue, P., Benincori, T., Bonetti, G., Cirilli, R., Bichon, S., Gounel, S., Mano, N., et al. (2021). Direct dynamic read-out of molecular chirality with autonomous enzyme-driven swimmers. Nat. Chem. *13*, 1241–1247.
18. Fosdick, S.E., Knust, K.N., Scida, K., and Crooks, R.M. (2013). Bipolar electrochemistry. Angew. Chem. Int. Ed. Engl. *52*, 10438–10456.

19. Karimian, N., Hashemi, P., Afkhami, A., and Bagheri, H. (2019). The principles of bipolar electrochemistry and its electroanalysis applications. Curr. Opin. Electrochem. *17*, 30–37.

20. Shida, N., Zhou, Y., and Inagi, S. (2019). Bipolar electrochemistry: a powerful tool for electrifying functional material synthesis. Acc. Chem. Res. *52*, 2598–2608.

21. Rahn, K.L., and Anand, R.K. (2021). Recent advancements in bipolar electrochemical methods of analysis. Anal. Chem. *93*, 103–123.

22. Koefoed, L., Pedersen, S.U., and Daasbjerg, K. (2017). Bipolar electrochemistry—a wireless approach for electrode reactions. Curr. Opin. Electrochem. *2*, 13–17.

23. Bouffier, L., Zigah, D., Sojic, N., and Kuhn, A. (2021). Bipolar (bio)electroanalysis. Annu. Rev. Anal. Chem. *14*, 65–86.

24. Sequeira, C.A.C., Cardoso, D.S.P., and Gameiro, M.L.F. (2016). Bipolar electrochemistry, a focal point of future research. Chem. Eng. Commun. *203*, 1001–1008.

25. Loget, G., and Kuhn, A. (2012). Bipolar electrochemistry in the nanosciences. Electrochemistry *11*, 71–103.

26. Cacucciolo, V., Shintake, J., Kuwajima, Y., Maeda, S., Floreano, D., and Shea, H. (2019). Stretchable pumps for soft machines. Nature *572*, 516–519.

27. Hann, S.Y., Cui, H., Nowicki, M., and Zhang, L.G. (2020). 4D printing soft robotics for biomedical applications. Addit. Manuf. *36*, 101567.

28. Salinas, G., Arnaboldi, S., Bouffier, L., and Kuhn, A. (2022). Recent advances in bipolar electrochemistry with conducting polymers. ChemElectroChem *9*, e202101234.

29. Mohammed, M., Thurgood, P., Gilliam, C., Nguyen, N., Pirogova, E., Peter, K., Khoshmanesh, K., and Baratchi, S. (2019). Studying the response of aortic endothelial cells under pulsatile flow using a compact microfluidic system. Anal. Chem. *91*, 12077–12084.

30. Sideris, E.A., and De Lange, H.C. (2020). Pumps operated by solid-state electromechanical smart material actuators - a review. Sens. Actuators A *307*, 111915.

31. Uvarov, I.V., Shlepakov, P.S., Melenev, A.E., Ma, K., Svetovoy, V.B., and Krijnen, G.J.M. (2021). A peristaltic micropump based on the fast electrochemical actuator: design, fabrication, and preliminary testing. Actuators *10*, 62.

32. Yan, B., Li, B., Kunecke, F., Gu, Z., and Guo, L. (2015). Polypyrrole-based implantable electroactive pump for controlled drug microinjection. ACS Appl. Mater. Interfaces *7*, 14563–14568.

33. Ebbens, S.J., and Howse, J.R. (2010). In pursuit of propulsion at the nanoscale. Soft Matter *6*, 726–738.

34. Ramırez-Garcıa, S., and Diamond, D. (2007). Biomimetic, low power pumps based on soft actuators. Sens. Actuators A *135*, 229–235.

35. Sannicolò, F., Mussini, P.R., Benincori, T., Cirilli, R., Abbate, S., Arnaboldi, S., Casolo, S., Castiglioni, E., Longhi, G., Martinazzo, R., et al. (2014). Inherently chiral macrocyclic oligothiophenes: easily accessible electrosensitive cavities with outstanding enantioselection performances. Chemistry *20*, 15298–15302.

36. Sannicolò, F., Arnaboldi, S., Benincori, T., Bonometti, V., Cirilli, R., Dunsch, L., Kutner, W., Longhi, G., Mussini, P.R., Panigati, M., et al. (2014). Potential-driven chirality manifestations and impressive enantioselectivity by inherently chiral electroactive organic films. Angew. Chem. Int. Ed. Engl. *53*, 2623–2627.

37. Arnaboldi, S., Vigo, D., Longhi, M., Orsini, F., Riva, S., Grecchi, S., Giacovelli, E., Guglielmi, V., Cirilli, R., Longhi, G., et al. (2019). Self-standing membranes consisting of inherently chiral electroactive oligomers: electrosynthesis, characterization and preliminary tests in potentiometric setups. ChemElectroChem *6*, 4204–4214.

38. Agrawal, K. (2007). Doxorubicin, xPharm: The Comprehensive Pharmacology Reference (Elsevier), pp. 1–5.

39. Komorsky-Lovrić, S. (2006). Redox kinetics of adriamycin adsorbed on the surface of graphite and mercury electrodes. Bioelectrochemistry *69*, 82–87.

40. Oliveira-Brett, A.M., Vivan, M., Fernandes, I.R., and Piedade, J.A.P. (2002). Electrochemical detection of in situ adriamycin oxidative damage to DNA. Talanta *56*, 959–970.

41. Oliveira-Brett, A.M., Piedade, J.A.P., and Chiorcea, A.-M. (2002). Anodic voltammetry and AFM imaging of picomoles of adriamycin adsorbed onto carbon surfaces. J. Electroanal. Chem. *538–539*, 267–276.

42. Yang, X., Zhang, X., Liu, Z., Ma, Y., Huang, Y., and Chen, Y. (2008). High-efficiency loading and controlled release of doxorubicin hydrochloride on graphene oxide. J. Phys. Chem. C *112*, 17554–17558.

43. Gupta, B., Zhang, L., Melvin, A.A., Goudeau, B., Bouffier, L., and Kuhn, A. (2020). Designing tubular conducting polymer actuators for wireless electropumping. Chem. Sci. *12*, 2071–2077.

44. Li, Y., and Qian, R. (2000). Electrochemical overoxidation of conducting polypyrrole nitrate film in aqueous solutions. Electrochim. Acta *45*, 1727–1731.

45. Eddin, L.B., Jha, N.K., Meeran, M.F.N., Kesari, K.K., Beiram, R., and Ojha, S. (2021). Neuroprotective potential of limonene and limonene containing natural products. Molecules *26*, 4535.

46. Bouyahya, A., Mechchate, H., Benali, T., Ghchime, R., Charfi, S., Balahbib, A., Burkov, P., Shariati, M.A., Lorenzo, J.M., and Omari, N.E. (2021). Health benefits and pharmacological properties of carvone. Biomolecules *11*, 1803.

Chem, Volume *10*

Supplemental information

Miniaturized enantioselective tubular devices for the electromechanical wireless separation of chiral analytes

Sara Grecchi, Gerardo Salinas, Roberto Cirilli, Tiziana Benincori, Sara Ghirardi, Alexander Kuhn, and Serena Arnaboldi

Supplemental Information

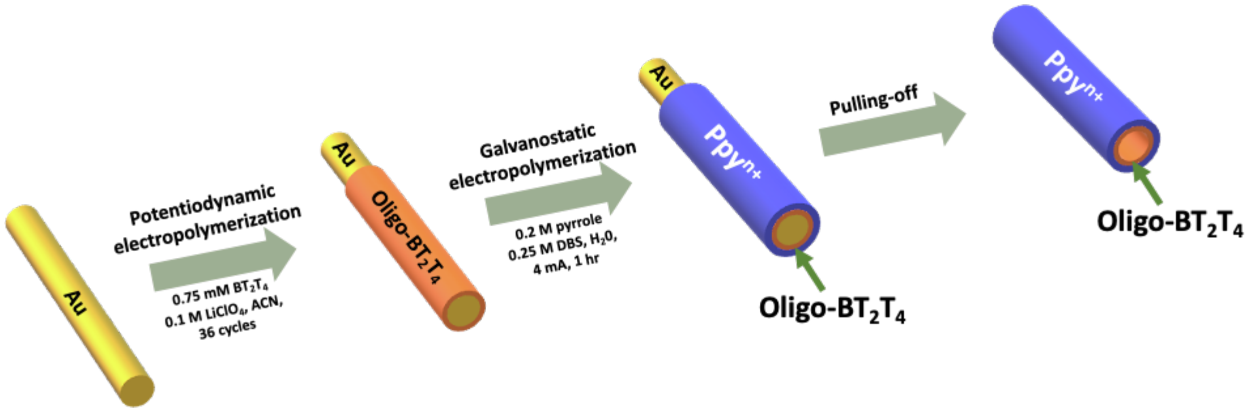

Figure S1. **Schematic illustration of the design of enantioselective tubes.** Schematic illustration of the fabrication steps of the polypyrrole-chiral oligomer tube.

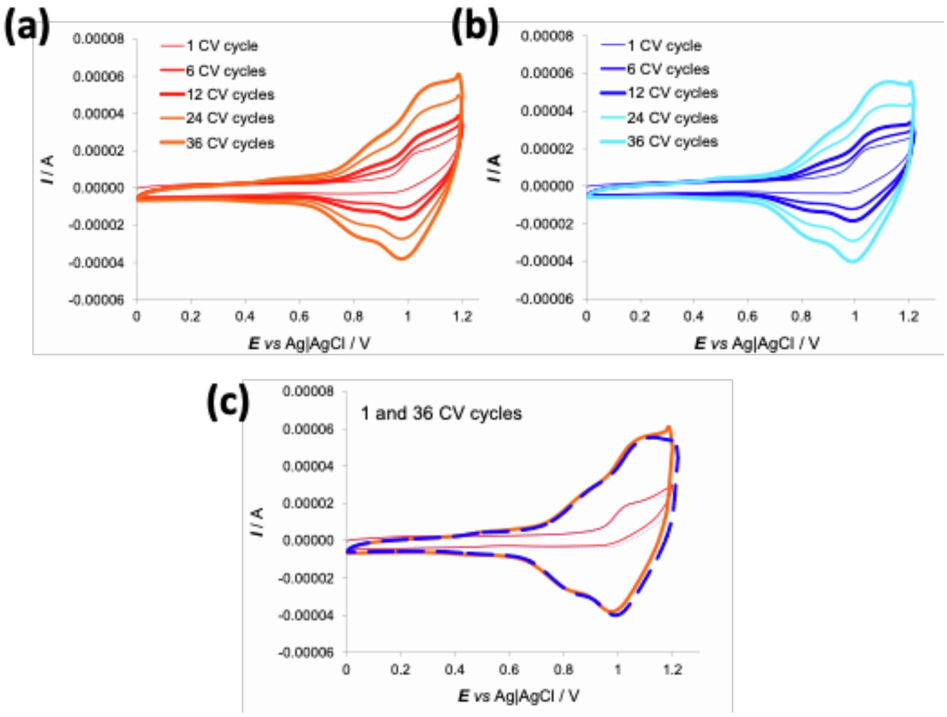

Figure S2. Electrooligomerization of (*S*)- and (*R*)-BT_2T_4. Potentiodynamic electrooligomerization of (a) (*S*)- and (b) (*R*)-BT_2T_4, in red and blue respectively (0. 75 mM in ACN + 0.1 M $LiClO_4$) on a GC electrode by potential cycling with 0.2 V/s. (c) Combined potentiodynamic plots of the 1st and 36th cycle of each monomer.

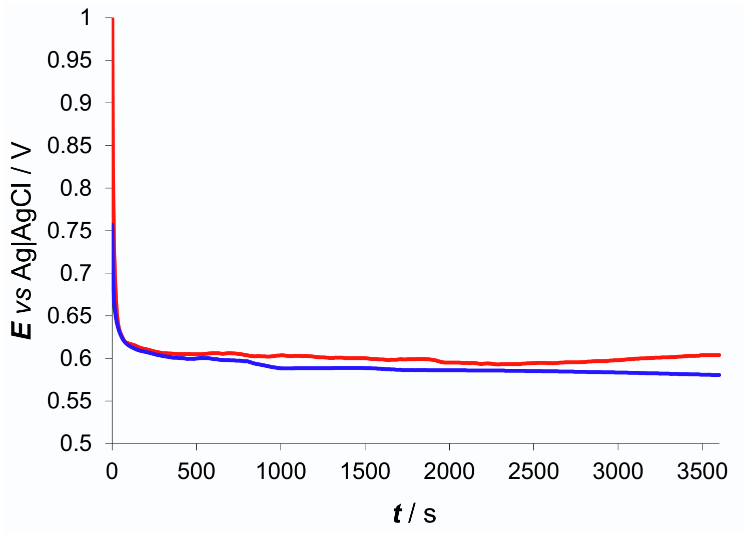

Figure S3. Galvanostatic electropolymerization of pyrrole on a gold wire modified with oligo-(*S*)- or (*R*)-BT_2T_4. Galvanostatic electropolymerization of pyrrole on a gold wire modified with oligo-(*S*)- or (*R*)-BT_2T_4 (in red and blue respectively), 400 μA, 3600 s.

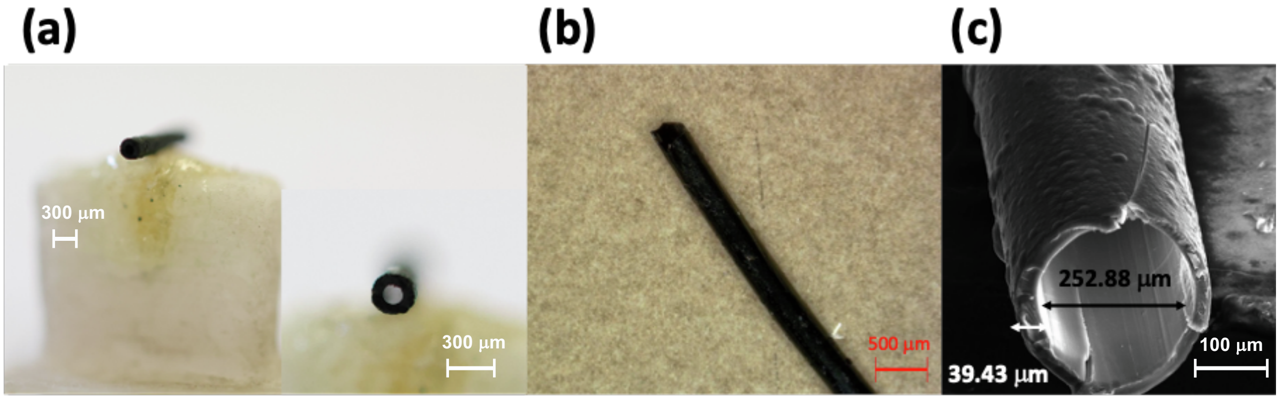

Figure S4. Optical characterization. Optical pictures of the soft tubes; (a) front view when positioned at the top of a plastic holder (scale bars 300 μm) and (b) top view (scale bar 500 μm). (c) SEM micrograph of the front view of one extremity of an unmodified tube. Scale bar 100 μm.

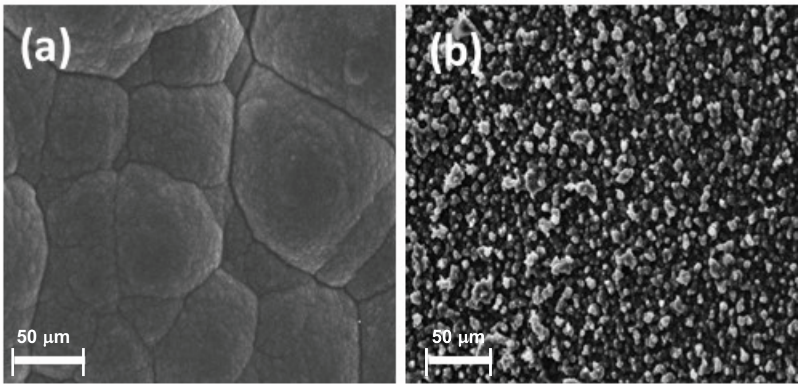

Figure S5. Morphological characterization. SEM micrographs of the a) external part and b) internal part of the tube, obtained cutting the functionalized tube along its main axis. Scale bars 50 μm.

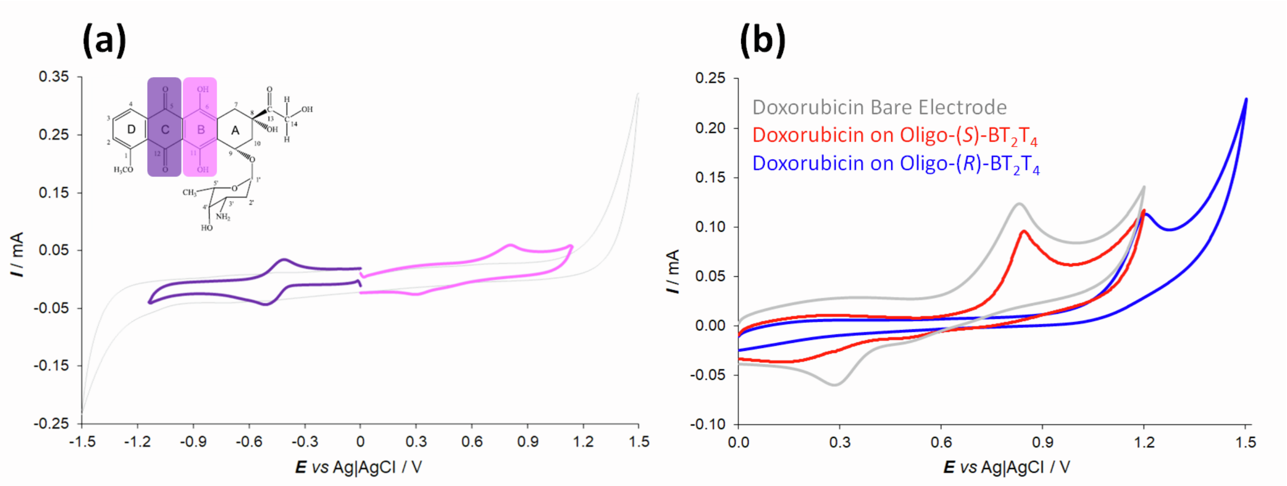

Figure S6. **Electrochemical characterization and enantioselectivity tests of doxorubicin.** (a) First oxidation and reduction peaks (right and left, respectively) of doxorubicin (1 mM in commercial buffer pH 4) on a GC electrode, at 0.2 V s^{-1} potential scan rate. The grey line stands for the background (commercial buffer pH4) measured under the same conditions. Oxidative and reductive half cycles are considered separately to avoid electrode poisoning by electron transfer products. (b) Enantioselectivity tests carried out in a pH 4 buffer solution containing 2 mM doxorubicin with oligo-(*S*)-BT_2T_4 or oligo-(*R*)-BT_2T_4 potentiodynamically deposited on a GC electrode. In grey, the doxorubicin signal recorded with a bare GC electrode.

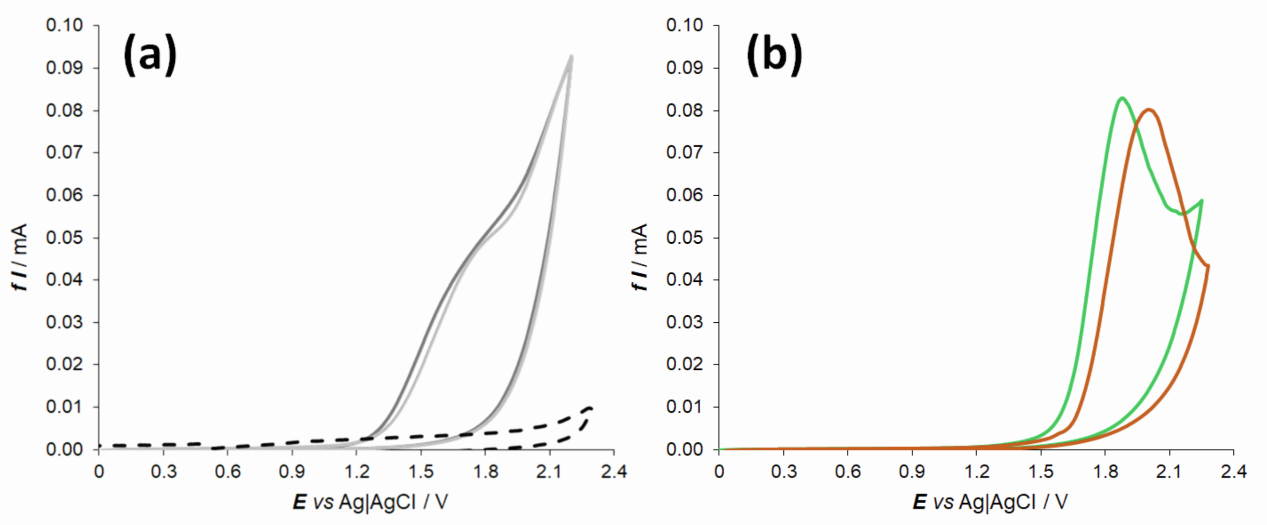

Figure S7. Enantioselectivity test with Carvone on (*R*)-oligo-BT_2T_4. (a) CV patterns recorded on a GC bare electrode at 50 mV/s potential scan rate of (*S*)-(+)- and (*R*)-(-)-carvone. The dotted black line stands for the background recorded in pH 4 buffer solution + EtOH. (b) Enantioselectivity tests in pH 4 buffer solution + EtOH at 50 mV/s potential scan rate of (*S*)-(+)- and (*R*)-(-)-carvone (2 mM, brown and green lines, respectively) on a GC electrode modified with oligo-(*R*)-BT_2T_4.

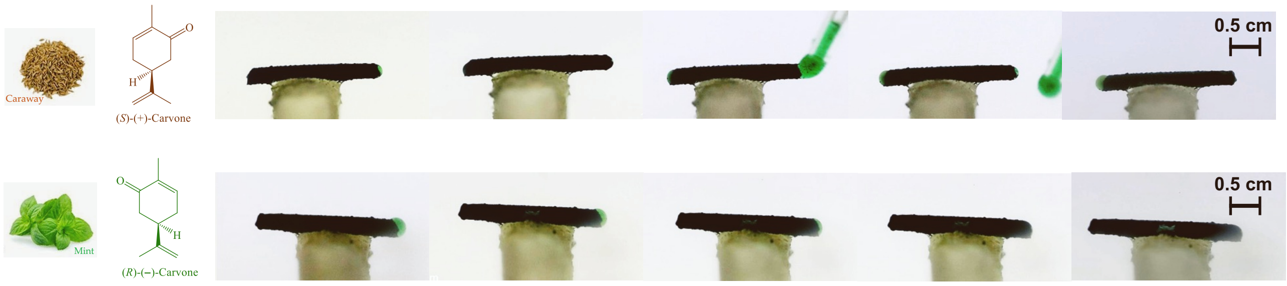

Figure S8. Enantioselective bipolar electrochemistry experiments with Carvone on (*R*)-oligo-BT_2T_4. Specular enantioselective bipolar electrochemistry experiments with (*S*)- and (*R*)-carvone (2 mM) dissolved in BMIMTFSI and chiral tubes modified with oligo-(*R*)-BT_2T_4. The total time for each acquisition was 10 minutes, optical pictures were taken every 2 minutes. Scale bars 0.5 cm.
